# Beta-2-transferrin to detect cerebrospinal fluid pleural effusion: a case report

**DOI:** 10.1186/1752-1947-3-6495

**Published:** 2009-03-13

**Authors:** Jennifer C Smith, Eyal Cohen

**Affiliations:** 1 Division of Pediatric Medicine, Department of Paediatrics, The Hospital for Sick Children, University of Toronto, Toronto, Canada

## Abstract

**Introduction:**

Pleural effusion secondary to ventriculoperitoneal shunt insertion is a rare and potentially life-threatening occurrence.

**Case presentation:**

We describe a 14-month-old Caucasian boy who had a ventriculoperitoneal shunt inserted for progressive hydrocephalus of unknown etiology. Two and a half months post-shunt insertion, the patient presented with mild respiratory distress. A chest radiograph revealed a large right pleural effusion and a shunt series demonstrated an appropriately placed distal catheter tip. A subsequent abdominal ultrasound revealed marked ascites. Fluid drained via tube thoracostomy was sent for beta-2-transferrin electrophoresis. A positive test was highly suggestive of cerebral spinal fluid hydrothorax. Post-externalization of the ventriculoperitoneal shunt, the ascites and pleural effusion resolved.

**Conclusion:**

Testing for beta-2-transferrin protein in pleural fluid may serve as a useful technique for diagnosing cerebrospinal fluid hydrothorax in patients with ventriculoperitoneal shunts.

## Introduction

Mechanical shunting of cerebrospinal fluid (CSF) is an effective treatment for non-obstructive hydrocephalus. In particular, ventriculoperitoneal (VP) shunting has become a preferred method in most clinical centers. Despite the wide acceptance of VP shunting, there are important complications associated with this technique. Common problems include obstruction, mechanical shunt failure and infections [1] while CSF ascites and thoracic complications, such as CSF hydrothorax, are less frequently observed sequelae [2]. Most cases of the lattermost complication, CSF hydrothorax, occur secondary to intrathoracic shunt tip migration [3]. However, a small number of cases, mostly in the pediatric population and secondary to massive ascites, have been reported with normal shunt position [2].

We report a 14-month-old Caucasian boy with a symptomatic pleural effusion that developed two and a half months post-VP shunt insertion for hydrocephalus of unknown etiology. We will review potential mechanisms behind the development of this complication and discuss the use of beta-2-transferrin for the identification of CSF hydrothorax in children with VP shunts.

## Case presentation

A 14-month-old Caucasian boy with idiopathic non-obstructive hydrocephalus and a VP shunt presented to the emergency room with a 1-day history of mild respiratory distress without cough or fever. The patient had presented at 11 months of age with a history of enlarging head circumference and developmental delay. A computed tomography (CT) scan of his head at 11 months of age revealed enlargement of the lateral, third and fourth ventricles and a bulky choroid plexus. A right-sided programmable valve VP shunt was inserted at that time.

On examination, the 14-month-old patient was found to be afebrile and tachypneic (34 breaths/minute) with mildly increased work of breathing and decreased respiratory sounds in the distal aspect of the right lung. The cardiovascular exam was within normal limits. An examination of the abdomen revealed distension but no hepatomegaly or signs of peritoneal irritation.

**Figure 1:**
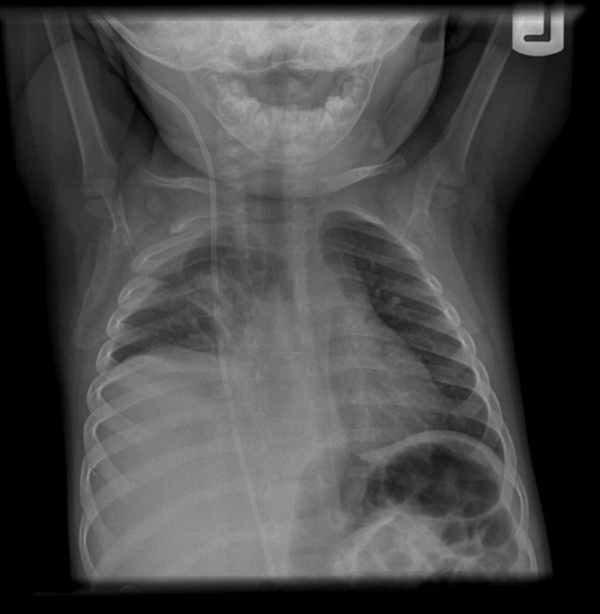
**Anteroposterior radiograph demonstrating a moderate to large right-sided pleural fluid collection which is predominantly subpulmonic in location**.

**Figure 2:**
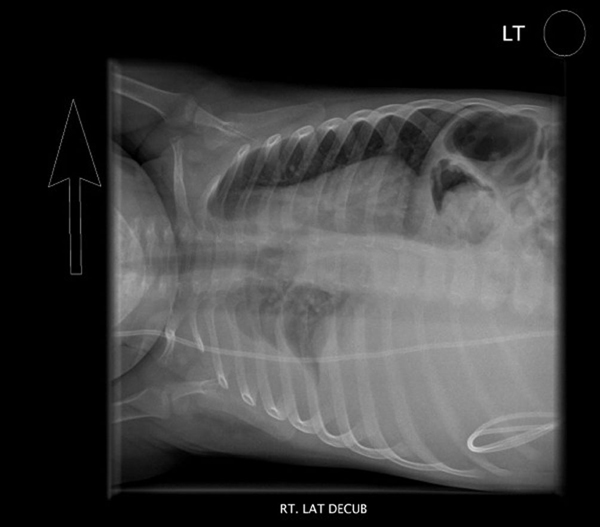
**Right lateral decubitus radiograph demonstrating a moderate to large right-sided pleural fluid collection which is predominantly subpulmonic in location**.

A chest radiograph (Figures 1 and 2) revealed a large right-sided pleural effusion, which was confirmed by chest ultrasonography to be a fluid collection measuring 12.3 × 9.2cm. A shunt series demonstrated the VP shunt to be in place without signs of discontinuity or leakage.

At the time of admission, venous blood gas, serum electrolytes and creatinine were within normal limits. Blood urea nitrogen and white blood count were mildly elevated at 5.1mmol/L and 12.5 × 10^9^/L, respectively. Further blood work demonstrated an alanine aminotransferase (ALT) level of 46U/L, an aspartate aminotransferase (AST) level of 51U/L, a serum calcium level of 2.61mmol/L and an alkaline phosphatase (ALP) level of 1403U/L. Subsequently, ALP steadily decreased to 498U/L but remained elevated throughout the patient's admission.

A thoracentesis was performed and a chest tube was inserted which drained >300cc/day of clear, yellow fluid. Around the time of chest tube insertion, the patient was noted to have an increasing abdominal girth, sizeable positive fluid balance and weight gain. An abdominal/pelvic ultrasound revealed marked ascites. Structurally normal major abdominal and pelvic viscera as well as normal vena caval and hepatic venous flows were reported.

A head CT showed no change from a previous scan carried out at 11 months of age. A parallel pleural fluid and CSF analysis was also performed which is detailed in Table 1. Secondary to the discordance between the white cell counts of the CSF and pleural fluid analyses, a sample of pleural fluid was sent for *β*_2_-transferrin assay and was found to be positive. Subsequent to this finding, the VP shunt was externalized followed by a dramatic decrease in chest tube drainage. A repeat chest radiograph revealed resolution of the pleural fluid collection and the chest tube was removed. The patient's abdominal girth decreased and a repeat abdominal/pelvic ultrasound demonstrated resolution of ascites. The patient's externalized ventricular drain was subsequently converted to a ventriculoarterial shunt.

**Table 1:** Pleural and cerebrospinal fluid analysis

Biochemical measure	Pleural fluid	Cerebrospinal fluid
Glucose	5.1g/L	4.1g/L
Total protein	≤10g/L	<0.1g/L
Triglyceride	<0.11g/L	
White blood count	440	38
Red blood count	20	0
Lymphocytes	50	56
Mesothelial cells	13	
Gram stain & culture	No organisms seen; sterile fluid	No organisms seen; sterile fluid

## Discussion

We report the use of *β*_2_-transferrin for the diagnosis of CSF hydrothorax in a child with a transudative pleural effusion. Although used previously in other applications, to the best of our knowledge, this is the first reported use of *β*_2_-transferrin to detect a CSF pleural effusion in a child with a VP shunt.

A variety of techniques to investigate ascites and/or pleural effusion in patients with VP shunts have been reported in the literature. Among the described methods is the use of radiopaque contrast. This particular procedure involves the injection of radioactive dye at a point along the shunt pathway followed by subsequent imaging studies to trace the migration of contrast-injected CSF [4]. An alternative diagnostic strategy in cases of CSF ascites or hydrothorax involves repositioning of the distal shunt, usually through relocation of the catheter tip within the peritoneal cavity or into the right atrium, with subsequent resolution of CSF fluid collections [5].

A novel diagnostic strategy was utilized in the case of our patient. A sample of fluid, drained via tube thoracostomy, was sent for beta-2-transferrin level. This desialated isoform of transferrin is almost exclusively found in the CSF with only minimal amounts present in cochlear perilymph and in the aqueous and vitreous humor of the eye. Multiple studies have validated the use of beta-2-transferrin as a specific marker for CSF leakage with sensitivity and specificity approaching 100% and 95%, respectively [6]. Furthermore, Huggins and Sahn (2003) report the use of beta-2-transferrin to identify the presence of a CSF pleural effusion in an elderly patient with a duro-pleural fistula [7]. To our knowledge, however, beta-2-transferrin has never been previously utilized to identity CSF hydrothorax in children with VP shunts.

Following externalization of the distal VP tip, our patient's ascites and pleural effusion promptly resolved. This finding, in combination with a positive beta-2-transferrin assay, was highly suggestive of a CSF hydrothorax.

The findings of CSF hydrothorax and ascites, as demonstrated in our patient, are rare complications of VP shunts. Reported to occur anywhere from days to years postoperatively, CSF ascites usually develops within the first 2 years after VP shunt placement [8]. In greater than 50% of these cases, clinical and/or laboratory investigations fail to reveal an underlying abdominal or other disease process to account for CSF ascites [9].

We propose a pathological intraperitoneal process, such as subclinical peritonitis, to account for the development of CSF ascites in our patient. Inflammation of the peritoneal membrane may lead to intraperitoneal accumulation of fluid through the impairment of lymphatic flow [9] and/or by increasing intra-abdominal pressure and volume [10].

As evidence to date suggests that modern shunt materials are inert, alternative sources of peritoneal irritation need to be considered. For example, an immune reaction to a foreign protein such as a vaccine may be enough to precipitate ascites in patients with VP shunts [5]. As the timing of our patient's most recent immunization in relation to his presentation is unknown, this possibility cannot be eliminated. Furthermore, although our patient's CSF and pleural fluid cultures were sterile, it is not possible to definitely refute the presence of an infectious process. For example, a subclinical viral infection may have been sufficient to cause peritoneal inflammation in our patient with resultant CSF ascites [11]. Furthermore, a strengthened immune response in infants, coupled with age-related decreased peritoneal absorptive capacity, makes subclinical peritonitis an important consideration in our patient [8].

Alternative explanations for CSF ascites in the literature such as excess CSF production, surgical procedures, and high CSF protein are less plausible explanations for our patient's CSF ascites. Firstly, it has been suggested that CSF ascites can result from excessive CSF production, with the excess fluid overwhelming the absorptive capacity of the peritoneum [12]. Despite an undiagnosed etiology for hydrocephalus in our patient, the absence of an interval change on CT scan makes augmented CSF overproduction an unlikely precipitant for his ascites. Secondly, a history of shunt revision or previous abdominal surgery has also been reported in patients with CSF ascites [5]. Although our patient underwent a shunt surgery two and a half months before presentation, there was no history of shunt revisions or abdominal surgeries.

Lastly, high CSF protein has been proposed as a potential cause of CSF ascites [9]. According to this supposition, an elevated CSF protein content may lead to reversed osmosis at the level of the blood-brain barrier with the resultant accumulation of excessive CSF fluid within the peritoneal cavity [13]. However, numerous cases of high CSF protein without resultant ascites have been reported [9]. Furthermore, as demonstrated in a previous case report, CSF ascites can occur despite normal levels of protein in the CSF [10].

The finding of CSF ascites in our patient can be used to explain the coincident finding of pleural effusion. Taub and Lavyne (2004) suggest three mechanisms to account for the development of CSF hydrothorax post-VP shunt placement [3]. Firstly, they propose that an error in surgical shunt placement, with resultant intrathoracic trauma, may account for some cases of CSF hydrothorax. Secondly, Taub and Lavyne suggest that pleural effusion may develop secondary to supra- or trans-diaphragmatic migration of the shunt tip into the thorax.

Considering that no surgical complications during VP shunt placement were reported in our patient and that his shunt series at the time of admission was normal, an alternative explanation is required. Thus, it is the third mechanism proposed by Taub and Lavyne, the development of hydrothorax secondary to CSF ascites that offers the most promising explanation for our patient's pleural effusion [3].

In order for CSF ascites to result in pleural effusion, some degree of open communication must exist between the peritoneal cavity and the pleural space. Congenital diaphragmatic defects or weak points in the diaphragm such as the Foramen of Morgagni or Foramen of Bochdalek provide potential conduits. Furthermore, one or more microscopic diaphragmatic communications is likely to be sufficient for the transdiaphragmatic movement of peritoneal fluid [2]. These posited congenital or acquired fenestrations enable peritoneal fluid to pass into the pleural space along a unidirectional pressure gradient [14]. This posited pressure differential results from a cyclic negative intrathoracic pressure, created during inspiration, coupled with increased hydrostatic intra-abdominal pressure [2]. In addition to causing a build-up of ascitic fluid, sub-clinical inflammation likely contributes to this process by facilitating the transudation of fluid through diaphragmatic capillary and lymphatic channels [4]. Fluid subsequently accumulates in the pleural space secondary to high volume transdiaphragmatic flow and the overwhelming of pleural absorptive abilities [14]. As the pleural surface remains pathology free with its sieve characteristics consequently intact, the cell and protein content of the ensuing pleural effusion should remain low [15]. These pleural fluid characteristics, along with a predominance of lymphocytes and mesothelial cells, suggest a transudative pleural effusion.

Although no gross diaphragmatic defect was identified in our patient, the presence of microscopic diaphragmatic communications may have been present. Notably, the tapped pleural fluid characteristics of our patient were found to be in keeping with a transudative effusion. The collected pleural fluid also demonstrated a marginally higher protein concentration than in the patient's CSF fluid. Differences in the solute composition of ventricular-extracted CSF and pleural fluid have been previously demonstrated in patients with pleural effusion secondary to VP shunts [2]. The finding of a slightly higher protein concentration in the pleural verses peritoneal fluid, the latter having been derived from CSF, can be explained by the preferential absorption of water over protein across the visceral pleura [15]. With this in mind, inconsistent solute characteristics, such as protein concentration, between pleural effusion and pure CSF should not be used to refute the possibility of CSF hydrothorax in patients with VP shunts.

## Conclusion

In summary, we present the case of a 14-month-old boy with CSF hydrothorax secondary to VP shunt. Pleural effusion following VP shunt insertion is a rare and potentially life-threatening event and hence requires prompt diagnosis and correction. We report a novel and non-invasive technique by which to identify the presence of CSF in pleural fluid. Beta-2-transferrin assay may serve as a useful new means for the identification of CSF hydrothorax in the context of patients with VP shunts.

## Consent

Written informed consent was obtained from the patient's mother for publication of this case report and any accompanying images. A copy of the written consent is available for review by the Editor-in-Chief of this journal.

## Competing interests

The authors declare that they have no competing interests.

## Authors' contributions

Both authors contributed to the conception of this case report. Dr. Jennifer Smith drafted the report while Dr. Eyal Cohen provided critical feedback. Both authors have reviewed and approve of the final version.
